# Ascorbic Acid, Inflammatory Cytokines (IL-1*β*/TNF-*α*/IFN-*γ*), or Their Combination's Effect on Stemness, Proliferation, and Differentiation of Gingival Mesenchymal Stem/Progenitor Cells

**DOI:** 10.1155/2020/8897138

**Published:** 2020-08-17

**Authors:** Karim M. Fawzy El-Sayed, Nhung Nguyen, Christof E. Dörfer

**Affiliations:** ^1^Stem Cells and Tissue Engineering Research Group, Faculty of Dentistry, Cairo University, Cairo, Egypt; ^2^Clinic for Conservative Dentistry and Periodontology, School of Dental Medicine, Christian Albrechts University, Kiel, Germany; ^3^Oral Medicine and Periodontology Department, Faculty of Dentistry, Cairo University, Egypt

## Abstract

**Objective:**

Ascorbic acid (AA) and controlled inflammatory stimuli are postulated to possess the ability to independently exert positive effects on a variety of proliferative, pluripotency, and differentiation attributes of gingival mesenchymal stem/progenitor cells (G-MSCs). The current study's objective was to explore and compare for the first time the impact of the major inflammatory cytokines (IL-1*β*/TNF-*α*/IFN-*γ*), AA, or their combination on multipotency/pluripotency, proliferative, and differentiation characteristics of G-MSCs.

**Design:**

Human G-MSCs (*n* = 5) were isolated and cultured in basic medium (control group), in basic medium with major inflammatory cytokines; 1 ng/ml IL-1*β*, 10 ng/ml TNF-*α*, and 100 ng/ml IFN-*γ* (inflammatory group), in basic medium with 250 *μ*mol/l AA (AA group) and in inflammatory medium supplemented by AA (inflammatory/AA group). All media were renewed three times per week. In stimulated G-MSCs intracellular *β*-catenin at 1 hour, pluripotency gene expression at 1, 3, and 5 days, as well as colony-forming units (CFUs) ability and cellular proliferation over 14 days were examined. Following a five-days stimulation in the designated groups, multilineage differentiation was assessed via qualitative and quantitative histochemistry as well as mRNA expression.

**Results:**

*β*-Catenin significantly decreased intracellularly in all experimental groups (*p* = 0.002, Friedman). AA group exhibited significantly higher cellular counts on days 3, 6, 7, and 13 (*p* < 0.05) and the highest CFUs at 14 days [median-CFUs (Q25/Q75); 40 (15/50), *p* = 0.043]. Significantly higher Nanog expression was noted in AA group [median gene-copies/PGK1 (Q25/Q75); 0.0006 (0.0002/0.0007), *p* < 0.01, Wilcoxon-signed-rank]. Significant multilineage differentiation abilities, especially into osteogenic and chondrogenic directions, were further evident in the AA group.

**Conclusions:**

AA stimulation enhances G-MSCs' stemness, proliferation, and differentiation properties, effects which are associated with a Wnt/*β*-catenin signaling pathway activation. Apart from initially boosting cellular metabolism as well as Sox2 and Oct4A pluripotency marker expression, inflammation appeared to attenuate these AA-induced positive effects. Current results reveal that for AA to exert its beneficial effects on G-MSCs' cellular attributes, it requires to act in an inflammation-free microenvironment.

## 1. Introduction

Periodontitis is an inflammatory disorder of tooth-investing and tooth-supporting tissues, branded by a gradual damage of alveolar bone, periodontal attachment, and eventually gingiva, associated with bacterial dysbiosis. The commencement of this multifaceted disease process commonly entails challenging of the periodontal immune-inflammatory system through virulent microbial biofilms. Subsequently, an inflammatory reaction is mounted, with the release of inflammatory cytokines, most prominently tumor necrosis factor alpha (TNF-*α*), interleukin (IL) 1 beta (IL-1*β*), IL-4, IL-6, and interferon gamma (IFN-*γ*) [1]. Duration and intensity of the resultant host reaction govern the personalized course and outcome of the inflammatory process, as well as affect the outcome of any subsequent periodontal reparative/regenerative approach.

Ascorbic acid (AA) is one of the pivotal biomolecule, with decisive effects on wound healing and collagen biosynthesis [2]. Investigations on adult [3, 4], embryonic [5], and induced pluripotent [6] stem/progenitor cells outlined the beneficial effects of AA on increasing cellular proliferation, impeding apoptosis, and triggering pluripotency markers' expression. Furthermore, in additional to its host modulatory effects in periodontal disease [2], it was suggested that AA supplementation could positively affect the outcome of periodontal reparative/regenerative therapies [7, 8].

Gingival mesenchymal stem/progenitor cells (G-MSCs) possess notable periodontal reparative and regenerative potentials [9, 10], with inflammation-resistance properties [11] and immunomodulation abilities in their local microenvironment [12]. The latest investigations outlined an individual G-MSCs-TLRs' expression profile [13] and defined two TLR-generated immunomodulatory phenotypes in G-MSCs challenged by TLR-agonists [14]. G-MSCs obtained from inflamed gingival tissues demonstrated a differentiation/regenerative aptitude comparable to G-MSCs from uninflamed tissues [15]. Furthermore, recent studies outlined positive short-term stimulatory effects of controlled inflammatory microenvironments on the G-MSCs' pluripotency, proliferation, and differentiation attributes [16–18]. Short-term inflammatory stimuli in isolation or combined with retinol supplementation boosted the stemness, proliferative, and differentiation capabilities of G-MSCs [19]. Recently, AA was shown to possess the ability to similarly enhance G-MSCs' proliferative aptitude and pluripotency marker expression [20]. The current study's aim is to investigate for the first time the AA and controlled inflammatory impacts, isolated or combined, on pluripotency, proliferation, Wnt/*β*-catenin pathway activation, and differentiation of G-MSCs.

## 2. Materials and Methods

### 2.1. Isolation/Characterization of G-MSCs

Human G-MSCs were obtained from healthy gingival collars (*n* = 5) at the Christian-Albrechts University of Kiel. The patient was taken as the experimental unit, and cells were not pooled. The present study was approved by the Ethics Committee of Christian-Albrechts University of Kiel (IRB D513/17). Cells' isolation, culturing in basic medium and immuno-magnetic cell sorting, employing anti-STRO-1 antibody (BioLegend, San Diego, CA, USA) and anti-IgM MicroBeads (MiltenyiBiotec, Bergisch Gladbach, Germany), was done as formerly described [19, 21]. Colony-forming units (CFUs) capability and the expression of the surface markers CD14, 34, 45, 73, 90, and 105 on second passage G-MSCs, using FACS Calibur E6370 and FACS Comp 5.1.1 software (Becton Dickinson), were further conducted as formerly described [16, 17, 21].

### 2.2. Multilineage Differentiation

- Osteogenic differentiation: 2 × 10^4^ second passage G-MSCs/well were cultivated in six-well plates in osteogenic inductive medium (PromoCell, Heidelberg, Germany) as well as basic medium (controls) for 21 days. Alizarin red (Sigma-Aldrich) staining of both cultures was done to assess calcified nodules formation and quantified
- Adipogenic differentiation: 3 × 10^5^ second passage G-MSCs/well were cultured in six-well culture plates in adipogenic inductive medium (PromoCell) as well as basic medium (controls) for 21 days. Oil-Red-O staining (Sigma-Aldrich) was used to examine the presence of intracellular lipid droplets and quantified
- Chondrogenic differentiation: 3D micromasses of second passage 3 × 10^4^ G-MSCs/tube were cultured in chondrogenic induction medium (PromoCell) and in basic medium (controls) (both in Eppendorf tubes, Eppendorf, Hamburg, Germany) for 35 days. Alcian blue and nuclear-fast-red counterstaining (Sigma-Aldrich) evaluated glycosaminoglycans' formation and quantified (all quantification methods are described below).

### 2.3. Experimental Groups

Second passage G-MSCs were cultured in basic medium (control group), in basic medium, with 1 ng/ml IL-1*β*, 10 ng/ml TNF-*α*, and 100 ng/ml IFN-*γ* (Pepro Tech Inc, Rocky Hill, NJ, USA) [13, 14, 16, 19, 22, 23] (inflammatory group), in basic medium and 250 *μ*g/ml AA [20] (AA group), or in inflammatory medium with 250 *μ*g/ml AA (inflammatory/AA group). Culture media were exchanged three times per week.

### 2.4. Intracellular Wnt/*β*-Catenin Evaluation by ELISA

Intracellular total *β*-catenin was measured (ELISA Kit, Invitrogen, CA, USA). 8 × 10^4^ G-MSCs per well were cultivated on a six-well plate and stimulated for 1 hour, according to the groups defined above, followed by PBS washing and 350 *μ*l lysis buffer addition. 50 *μ*l standard or sample was mixed with 100 *μ*l *β*-catenin (total) detection antibody and incubated for one hour at room temperature. After addition of 100 *μ*l of Ant-Rabbit IgG HRP Working Solution, shaking the plates for 30 minutes, 100 *μ*l of Stabilized Chromogen was supplemented for 30 minutes in the dark, followed by 100 *μ*l Stop Solution, and OD documented at 450 nm (MultiskanGO Microplate Spectrophotometer, Thermo Fisher, Langenselbold, Germany). Intracellular total *β*-catenin was determined using standard curves.

### 2.5. CFUs and Cellular Proliferation

1 × 10^4^ second passage G-MSCs per well of each experimental group were cultivated in 24-well culture plates, and their cellular counts were determined every day for 14 days.

MTT (3-[4,5-dimethylthiazol-2-yl]-2,5-diphenyl-tetrazoliumbromide) test was conducted at 24 and 72 hours (MTT Cell Proliferation Kit-I, Roche Diagnostics GmbH, Mannheim, Germany) [24] to test the cells metabolic activity. No phenol-red serum-free medium (RPMI 1640, PAN-Biotech, Aidenbach, Germany) and 0.5 mg/ml MTT-labelling reagent was added to the G-MSCs cultures and left for 4 hours, followed by 1 ml of the Solubilization solution (37°C, 5% CO_2_, overnight). The spectrophotometrical absorbance was recorded at 570 nm wavelength (MultiskanGO Microplate Spectrophotometer, Thermo Fisher). Metabolic activity was calculated using standard curves. The assays were conducted in duplicate and averaged.

Second passage 1.63/cm^2^ G-MSCs were cultivated in 10-cm-diameter dishes for the CFUs assay. On the 14^th^ day, cell cultures were fixed using 100% methanol (ice-cold, for 10 min) and stained with 0.1% crystal violet for 10 min. CFUs were evaluated by two independent examiners, using phase-contrast inverted microscopy. Aggregations of ≥50 cells were counted as a colony.

### 2.6. G-MSCs' mRNA Expression

To test for the pluripotency gene expressions (Nanog, octamer-binding-transcription-factor 4A (Oct4A) as well as sex-determining-region-Y-box 2 (Sox2)), mRNA extraction was done in the four experimental groups outlined above at 24, 72, and 120 hours, using the RNeasy kit (Qiagen, Hilden, Germany). Complementary DNA (cDNA) was synthesized from RNA (1 *μ*g/*μ*l) via reverse transcription (QuantiTect reverse transcription kit, Qiagen) in a volume of 20 *μ*l reaction mixture (4 pmol of each primer, 10 *μ*l of the LightCycler Probes Master mixture (Roche) and 5 *μ*l specimen cDNA). Real-time polymerase chain reaction (rt-PCR; LightCycler 96 Real-Time PCR System, Roche Molecular Biochemicals, Indianapolis, Indiana, USA) was conducted. Nineteen possible reference genes were preexamined to decide on the most suitable reference gene in the G-MSCs, which would not be regulated by the experiment (NormFinder). Except for PGK1, all were regulated. Hence, PGK1 (housekeeping gene) was determined to be employed as a reference gene (Table 1). The relative quantification of the examined genes was conducted employing the 2^-*ΔΔ*Ct^ method and assays done in triplicate and averaged. Gene expressions of each of the tested genes were normalized to PGK1.

### 2.7. Multilineage Potential of Stimulated G-MSCs

G-MSCs were prestimulated for five days in the experimental groups designated above, followed by osteogenic (21 days), adipogenic (21 days), or chondrogenic (35 days) differentiation. mRNA expression of alkaline phosphatase (ALP) and Runt-related transcription factor 2 (Runx2), as well as the qualitative and quantitative Alizarin red staining, was conducted to assess the G-MSCs' osteogenic aptitude. For the quantification of the Alizarin red staining, 200 *μ*l 10% acetic acid was supplemented into the osteogenically induced G-MSCs cultures for 30 minutes on a shaker, followed by detachment of the cellular monolayer, and transferred into a 1.5 ml tubes (Eppendorf) and vortexing for 30 seconds. Following 10 minutes 85°C heating and ice-cooling, the mixture was centrifuged at 20,000 rpm (15 minutes). 200 *μ*l supernatant was transported to a new 1.5-ml tube and the pH neutralized with 10% ammonium hydroxide. Spectrophotometrical absorbance of 50 *μ*l of the sample and Alizarin red standards were recorded at OD405 (Thermo Fisher), and relative Alizarin red quantities were determined [25].

mRNA expression for lipoprotein lipase (LPL) and proliferator-activated receptor gamma (PPAR-*γ*) and quantitative and qualitative Oil-Red-O evaluation were assessed as evidence for the adipogenic differentiation aptitude. For Oil-Red-O quantification, isopropanol (1 ml/well) was added, and the cultures were incubated for 15 minutes on a shaker, and 100 *μ*l of the resultant mixture's spectrophotometrical absorbance was measured at OD540 (Thermo Fisher), and finally, the relative Oil-Red-O quantities were determined [26].

To assess chondrogenic differentiation, Aggrecan (ACAN) mRNA expression and Alcian blue/nuclear-fast-red staining were evaluated. For quantification of Alcian blue and nuclear-fast-red staining, automated digital image quantification of the chondrogenic differentiation was performed as previously described [27]. All primers used in the real-time PCR were supplied by Roche (Table 1).

### 2.8. Statistical Analysis

The normality of the data was evaluated, using the Shapiro-Wilk-test. Differences in intracellular total *β*-catenin, CFUs, MTT, mRNA expression, and quantitative adipogenic and osteogenic differentiation between the experimental groups were analyzed employing the Friedman-test. Differences in MTT and mRNA expression at different time points and pairwise comparisons were conducted using the Wilcoxon-signed-rank-test (SPSS 11.5, IBM, Chicago, IL, USA). The significance level was set at *p* = 0.05.

## 3. Results

### 3.1. Characterization of G-MSCs

**Figure 1:**
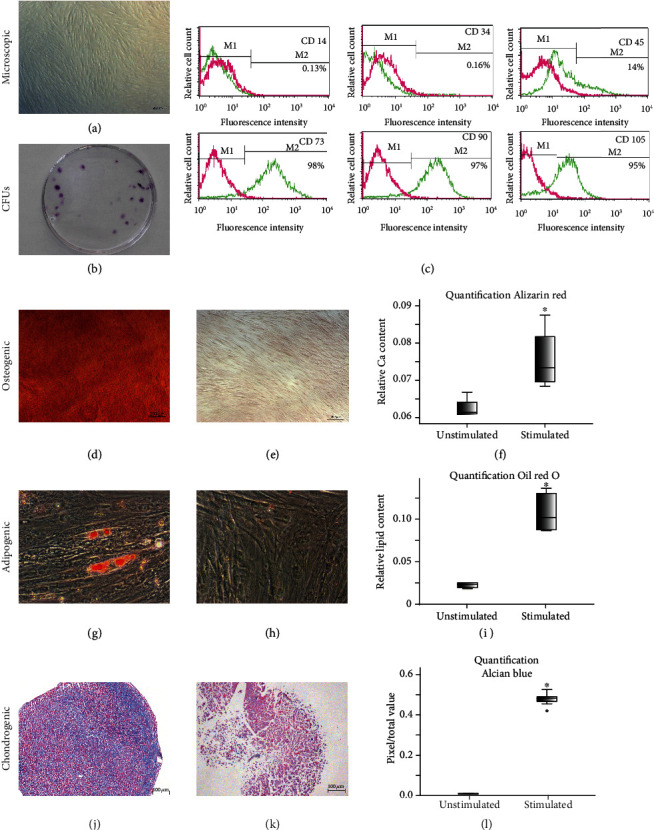
Phase contrast inverted microscopic picture of gingival cells growing out from a gingival connective tissue specimen (a). G-MSCs' colony-forming units (CFUs) (b). G-MSCs surface markers' expression flowcytometrically (c). Osteogenic induction of stimulated G-MSCs (Alizarin red staining; d) and respective unstimulated controls (e) with quantification (f). Adipogenic induction of stimulated G-MSCs (Oil-Red-O stained; g) and respective unstimulated controls (h) with quantification (i). Chondrogenic induction of stimulated G-MSCs (Alcian blue/acid-fast red staining; j) and respective unstimulated controls (k), with quantification (l). Significant differences denoted with asterisks (*n* = 5, ^∗^*p* < 0.05, Wilcoxon-signed-rank-test).

Following adhesion, cells grew out of the gingival tissues, demonstrating fibroblast-like morphology (Figure 1(a)). Characteristic CFUs were demonstrated by the G-MSCs (Figure 1(b)), which were negative for CD14, CD34, weakly positive for CD45, and highly positive for CD73, CD90, and CD105 surface markers expression (Figure 1(c)). Osteogenically induced G-MSCs formed significantly higher Alizarin red-labelled calcified deposits, as opposed to unstimulated controls (Figures 1(d)–1(f)). The G-MSCs' adipogenic differentiation formed significantly higher Oil-Red-O positively stained lipid inclusions, as opposed to unstimulated controls (Figures 1(g)–1(i)). Chondrogenically induced G-MSCs formed significantly higher Alcian blue-positive glycosaminoglycans deposits, as opposed to unstimulated controls (Figures 1(j)–1(l); *p* < 0.05, Wilcoxon-signed-rank-test).

### 3.2. The Intracellular Wnt/*β*-Catenin Pathway

**Figure 2:**
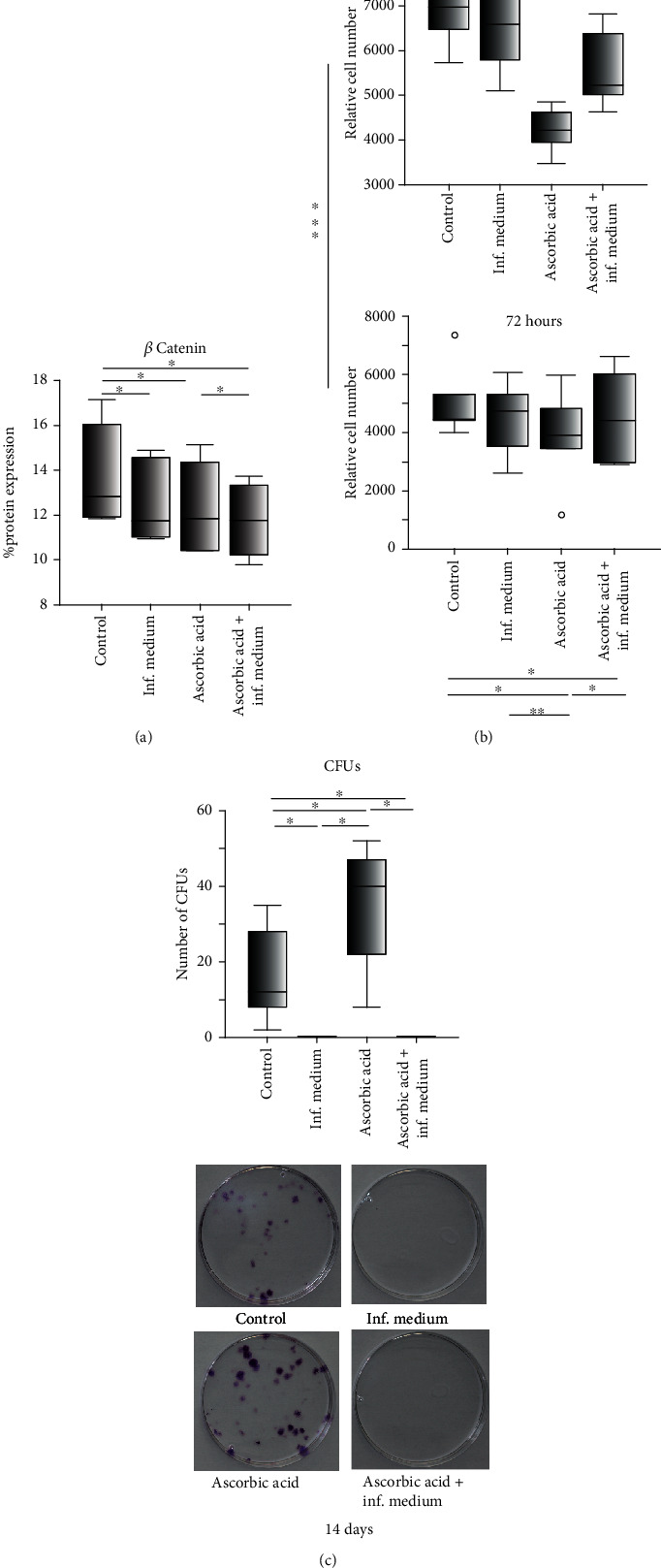
Wnt/*β*-catenin pathway activation, MTT, and CFUs following ascorbic acid and inflammatory stimulation of G-MSCs: ELISA examination of the Wnt/*β*-catenin signaling pathway for total intracellular *β*-catenin following G-MSCs' challenging by ascorbic acid and inflammation (a; box and whisker plots with medians/quartiles). Relative G-MSCs metabolic activity following ascorbic acid and inflammatory stimulation for 24 h and 72 h (b; box and whisker plots with medians/quartiles). CFUs-assay/CFUs' numbers following G-MSCs' stimulation via ascorbic acid and inflammation (c; box and whisker plots with medians/quartiles). Significant differences denoted with asterisks (*n* = 5, ^∗^*p* < 0.05; ^∗∗^*p* < 0.01; Wilcoxon-signed-rank-test). Abbreviations: CFUs: colony-forming units; *β*-catenin: total *β*-catenin.

G-MSCs' intracellular Wnt/*β*-catenin pathway was activated by AA and inflammation (Figure 2(a)), significantly reducing the total intracellular *β*-catenin, as compared to the control group*. β*-Catenin levels intracellularly were the greatest (median% expression, Q25/Q75) in control group (12.8%, 11.9/16.3), trailed by AA group (11.8%, 10.4/14.6), inflammatory/AA group (11.8%, 10.1/13.4), and finally the inflammatory group (11.7%, 11.0/14.6; *p* = 0.002, Friedman).

### 3.3. CFUs and Cellular Proliferation

Over 14 days, the greatest cellular counts were evident in AA group, trailed by inflammatory/AA group, inflammatory group, and finally control group (Table 2, Friedman). The highest cellular metabolic activity was demonstrated between 24 hours and 72 hours in the control (6109, 4457/7290), trailed by the inflammatory group (5558, 4444/6815), the inflammatory/AA group (5116, 4055/6434), and finally the AA group (4083, 3466/4845; *p* = 0.004). At 14 days, CFUs were the greatest (Median CFUs, Q25/Q75) in AA group (40, 15/50), trailed by control group (12, 5/32), and minute CFUs in inflammatory challenged groups (*p* = 0.002, Friedman, Figures 2(b) and 2(c)).

### 3.4. M-RNA Expression of Pluripotency Markers

**Figure 3:**
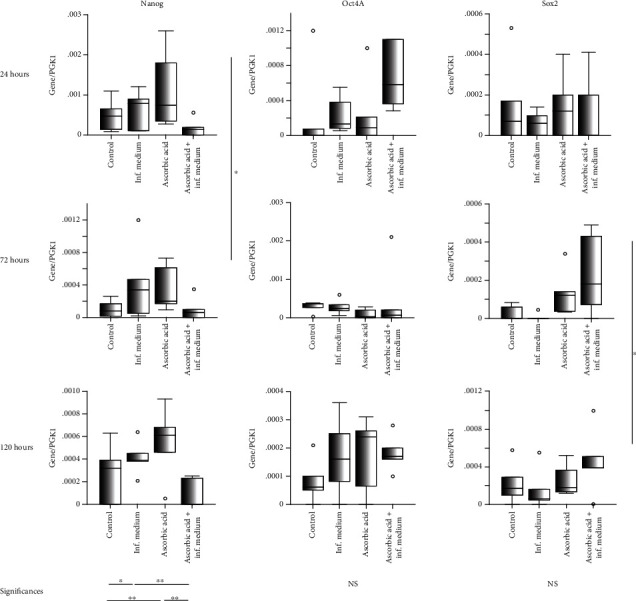
mRNA pluripotency genes expression (Nanog, Oct4A, Sox2) in G-MSCs challenged by ascorbic acid and inflammation at 24, 72, and 120 hours (box and whisker plots with medians/quartiles). Significant differences denoted with asterisks (*n* = 5, ^∗^*p* < 0.05, ^∗∗^*p* < 0.01; Wilcoxon-signed-rank-test). Abbreviations: Sox2: sex-determining region Y-box 2; Oct4A: octamer-binding-transcription-factor 4A; NS: nonsignificant.

Regarding pluripotency gene expressions (Figure 3), significantly, higher expressions for Sox2 were noted overtime (median gene copies/PGK-1, Q25/Q75) from (0.00006, 0.00000/0.00019) to (0.00041, 0.00000/0.00014; *p* = 0.04, Friedman). Significantly, higher expression of Nanog was noted in AA group (0.0006, 0.0002/0.0007), trailed by the inflammatory group (0.0004, 0.0001/0.008), the control group (0.0002, 0.0000/0.0005), and inflammatory/AA group (0.00006, 0.0000/0.0002; *p* < 0.001, Friedman). Overall, Oct4A and Sox2 were the highest expressed in the inflammatory/AA group ((0.0002, 0.0001/0.0006) and (0.0002, 0.0000/0.0004), respectively).

### 3.5. Stimulated G-MCSs' Multilineage Differentiation Potential

**Figure 4:**
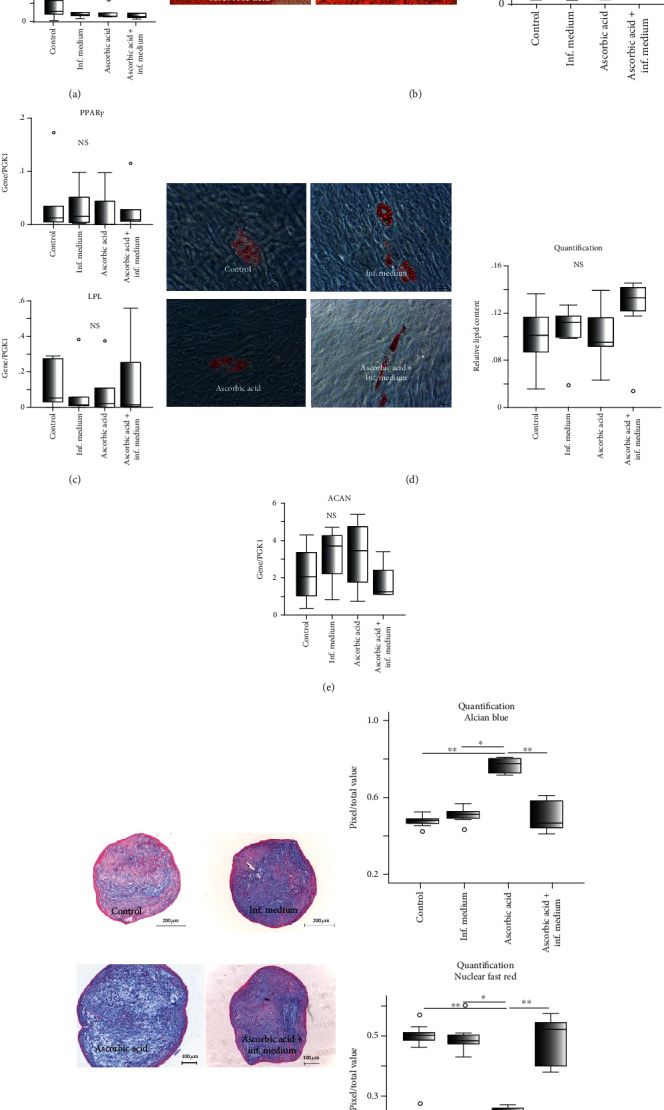
G-MSCs multilineage differentiation following stimulation by ascorbic acid and inflammation: gene expressions of ALP and RUNX2 following a 21-day osteogenic stimulation (a; box and whisker plots with medians/quartiles). Ca^2+^ quantification and Alizarin-Red staining following a 21-day osteogenic induction of ascorbic acid and inflammation stimulated G-MSCs (b; box and whisker plots with medians/quartiles). LPL and PPAR*γ* gene expression after 21 days of adipogenic stimulation of ascorbic acid and inflammation challenged G-MSCs (c; box and whisker plots with medians/quartiles). Oil-Red-O staining and lipid amount quantification of ascorbic acid and inflammation stimulated G-MSCs after 21 days of adipogenic stimulation (d; box and whisker plots with medians/quartiles). ACAN gene expression following a 35-day chondrogenic induction of ascorbic acid and inflammation stimulated G-MSCs (e; box and whisker plots with medians/quartiles). Alcian blue/nuclear-fast-red staining and their quantification in ascorbic acid and inflammation stimulated G-MSCs following a 35-day chondrogenic induction (f). Significant differences denoted with asterisks (*n* = 5, ^∗^*p* < 0.05, ^∗∗^*p* < 0.01; Wilcoxon-signed-rank-test). Abbreviations: ACAN: Aggrecan; ALP: alkaline phosphatase; LPL: lipoprotein lipase; NS: nonsignificant.

G-MSCs in the different experimental groups exhibited a remarkable multilineage differentiation aptitude (Figure 4). No significant differences were observed for RUNX2, ALP, LPL, PPAR-*γ*, or ACAN expression between the different groups in their respective inductive media. Histochemical staining of cellular cultures in the experimental groups exhibited calcified nodules after osteogenic induction, Oil-Red-O-positive lipid inclusions after adipogenic induction, and glycosaminoglycan deposition, following chondrogenic induction. For osteogenic and chondrogenic stimulation, significantly, the highest Alizarin red and Alcian blue with the lowest nuclear-fast-red staining was, respectively, evident in the AA group as compared to the other examined groups (*p* < 0.05, Wilcoxon-signed-rank-test).

## 4. Discussion

Reparative/regenerative approaches in periodontology are based principally on recapitulating the chief periodontal developmental events, encompassing stem/progenitor cells' proliferation, migration, homing, differentiation, and finally maturation [28]. Clinically, these healing stages primarily take place in an initially inflamed periodontal microenvironment, with inflammatory cytokines orchestrating the course of the inflammatory periodontal disease progression [29], possible healing/regeneration [30], as well as periodontal stem/progenitor cells' attributes [18, 19].

Apart from its important roles in periodontal wound healing, tissue regeneration [31], and collagen synthesis of bone, teeth, and gingiva, AA demonstrates potent cellular protective antioxidative properties in response to periodontitis-induced oxidative inflammatory reactions [32, 33]. It further possesses immunomodulatory abilities, which could markedly downregulate IL-1*α*, IL-1*β*, IL-6, tumor necrosis factor beta (TNF-*β*), and nitric oxide production in periodontal lesions [34, 35]. AA could further induce a pluripotent stage [36] and enhance the reprogramming efficiency [6] in embryonic stem cells.

Similar to previous investigations [21, 37–40], the tested G-MSCs showed all predefined stem/progenitor cells' hallmarks encompassing CFUs ability, surface markers' expression, and a remarkable multilineage differentiation capacity [41]. Recently, multiple investigations have revealed that local microenvironmental inflammatory stimuli could uplift G-MSCs' reparative and regenerative potentials [16–19, 42, 43]. The currently investigated G-MSCs were stimulated via periodontal proinflammatory cytokines, namely IL-1*β*, TNF-*α*, and IFN-*γ* [16], by AA or their combination. Subsequently, the G-MSCs' pluripotency, proliferation, and differentiation potentials were assessed. Challenging the G-MSCs' through AA and inflammatory stimuli appeared to activate their Wnt/*β*-catenin pathway, culminating in a significant reduction of their *β*-catenin levels intracellularly, possibly affecting their stemness [44], proliferative [45], and differentiation capacity [46].

Comparable to oral wound healing [47], periodontal wound healing undergoes five phases, namely a hemostatic phase, an inflammatory phase, a cellular recruitment and proliferation phase, and finally a tissue remodeling one. Hereby, G-MSCs rely primarily on their stemness in performing theses periodontal tissue reparative and regenerative actions, primarily proliferation and multilineage differentiation potential. The current study examined the effect of the inflammatory cytokines mixture together and in combination with AA on the pluripotency markers' expression, namely Nanog, Oct4A, and Sox2 at 24, 72, and 120 hours. In line with earlier studies, in the present one Nanog, Oct4A, and Sox2 expressions were amplified in the AA- [3, 20, 48] and inflammation-challenged [19, 49] G-MSCs. The AA-induced increase in the pluripotency markers Oct4A and Nanog could be ascribed primarily to the capability of AA, similar to Retinol [19], to activate the ten-eleven-translocation (TET) demethylases, eliminating methylation of the DNA and thereby inciting intracellular epigenetic reprogramming actions, encompassing pluripotency amplification [19, 50, 51] in G-MSCs. Yet, the current study further demonstrated their synergistic effect, with the highest upregulation of Oct4A and Sox2 expression being notable on the combination of AA with the inflammatory cocktail. It appears that a combination of AA and inflammation could augment their action on the expression of nuclear markers of pluripotency in the G-MSCs, thereby upregulating their stemness.

Cellular proliferation represents a primary requirement of any periodontal regenerative/reparative approach to obtain cellular counts able of executing the ensuing phases of migration/homing and functional tissue differentiation. A fundamental hallmarks of stem/progenitor cells remains to be their ability for colonogenic self-renewal, demonstrated through their CFUs producing potential. Inflammatory stimulation, in line with previous investigations [17], appeared to enhance G-MSCs' metabolic activity at 72 hours. Surprisingly, AA stimulation initially appeared to decrease cellular metabolic activity in the early phase and even diminish the augmenting effects of the inflammatory stimuli in the inflammatory/AA group. The observed attenuation of G-MSCs' metabolic activity, while inducing proliferation could be attributed to the earlier reported property of AA to suppress cellular growth arrest encoding genes, namely growth arrest/DNA-damage-inducible 45*α* (Gadd45a) as well as apoptosis-inducing genes, namely caspase-1 [35]. It further can be ascribed to the potential of AA to increase ERK1/2 phosphorylation with a concomitant attenuation of the mitogen-activated kinase pathway [52]. In the current study, AA exhibited over 14 days a proliferative-induction potential, which was previously reported in longer-term cultures [48, 53], and significantly higher CFUs formation. This property could be primarily attributed to an increase in the AA-mediated upregulation of the proliferation-related-Fos-transcriptional-factor [52]. In contrast, the tested inflammatory microenvironment drove the G-MSCs to self-senescence on lengthier stimulation [17]. Short-term inflammatory stimuli could induce cellular proliferation through Wnt/*β*-catenin activation and a subsequent subdual of the noncanonical Wnt/Ca^2+^ pathways [18, 45], which was evident by the reduced total intracellular *β*-catenin observed in the challenged groups. Yet on excessive stimulation, TNF-*α* could induce self-senescence of the stem/progenitor cells, especially in the presence of IFN-*γ* and cultivation at lower cellular densities, as in the CFU experiment. Through altering the IFN-*γ*-activated, nonapoptotic form of TNF-receptor-superfamily-member-6 (Fas) signaling into a caspase 3- and caspase 8-associated proapoptotic cascade, the G-MSCs' apoptotic pathway could have been activated [54]. AA and short-term controlled inflammation appear to have opposed biphasic impacts on the short-term as well as the long-term G-MSCs' proliferation.

AA is generally characterized by its ability to modulate cell growth, metabolism, and morphogenesis during osteogenesis [55, 56], as well as to induce extracellular matrix production [3]. AA stimuli boosted the G-MSCs' multilineage differentiation potentials, particularly osteogenic as well as chondrogenic differentiation capacity. In this context, the observed Wnt/*β*-catenin pathway activation, with a substantial reduction in the total *β*-catenin intracellularly plays an important role. An intracellular *β*-catenin accumulation, with a subsequent translocation to the nucleus and resultant stimulation of the lymphocyte enhancer-binding factor-1 (Lef-1), silences the RUNX2/Osterix-associated axis of osteogenesis [46]. Hence, the currently noted intracellular total *β*-catenin downregulation, aside from a proliferatory enhancement effect, would heighten the G-MSCs' differentiation capacity. Notably, the greatest multilineage differentiation capability was evident in the AA group, emphasizing the importance of an inflammation-free environment for a successful multilineage differentiation of G-MSCs boosted by AA.

Combined, current results point at enhanced G-MSCs' characteristics in the presence of AA, which, apart from an initially observed synergistic effect on cellular metabolism as well as Sox2 and Oct4A pluripotency markers expression, were attenuated in the presence of an inflammatory microenvironment. The observed effects appeared to be associated with a Wnt/*β*-catenin pathway activation. Similar to previous investigations, precise short-termed microenvironmental inflammatory stimuli could enhance early cellular attributes and pluripotency, while an AA-induced boosting of cellular proliferation and differentiation would require an inflammation-free microenvironment. The present results denote that an early short-termed controlled G-MSCs' inflammatory stimulation, followed by a AA stimulation in an inflammation-free microenvironment could provide an interesting scheme for enhancing their cellular attributes in regenerative approaches.

## Figures and Tables

**Table 1:** Real-time PCR primers (Roche).

Gene	Gene symbol	Accession ID	Assay ID
RUNX-2	RUNX2 H.sapiens	ENST00000359524	113380
ACAN	ACAN H.sapiens	ENST00000439576	138057
ALP	ALP H.sapiens	ENST00000374840	103448
LPL	LPL H.sapiens	ENST00000311322	113230
Nanog	Nanog H.sapiens	ENST00000229307	148147
Oct4A	Oct4 H.sapiens	ENST00000259915	113034
PGK-1	PGK1 H.sapiens	ENST00000373316	102083
PPAR*γ*	PPAR*γ* H.sapiens	ENST00000287820	110607
Sox2	Sox2 H.sapiens	ENST00000325404	111867

Abbreviations: ACAN: Aggrecan; ALP: alkaline phosphatase; LPL: lipoprotein lipase; Oct4A: octamer-binding-transcription-factor 4A; PGK-1: phosphoglycerate kinase-1; PPAR*γ*: proliferator-activated receptor gamma; RUNX-2: Runt-related transcription factor 2; Sox2: sex-determining region Y-box 2.

**Table 2:** Cellular counts in the control-, inflammation-, ascorbic acid- and inflammation/ascorbic acid-stimulated G-MSCs over 14 days (median and Q25/Q75 quartiles; Friedman test).

		Control	Inflammation	Ascorbic acid	Inflammation/ascorbic acid	*p* value
Day						
1	Median	1.83*E* + 04	1.33*E* + 04	1.83*E* + 04	1.58*E* + 04	0.33
	Q25	1.25*E* + 04	1.33*E* + 04	1.50*E* + 04	1.42*E* + 04	
	Q75	1.92*E* + 04	1.67*E* + 04	2.58*E* + 04	2.00*E* + 04	
2	Median	3.33*E* + 04	2.08*E* + 04	3.75*E* + 04	2.92*E* + 04	0.06
	Q25	2.50*E* + 04	1.58*E* + 04	2.75*E* + 04	2.67*E* + 04	
	Q75	3.92*E* + 04	2.25*E* + 04	4.17*E* + 04	3.50*E* + 04	
3	Median	4.42*E* + 04	3.50*E* + 04	5.25*E* + 04	3.92*E* + 04	0.04
	Q25	3.67*E* + 04	3.17*E* + 04	5.25*E* + 04	3.75*E* + 04	
	Q75	6.50*E* + 04	3.67*E* + 04	5.83*E* + 04	4.42*E* + 04	
4	Median	4.50*E* + 04	4.67*E* + 04	6.17*E* + 04	5.25*E* + 04	0.45
	Q25	3.58*E* + 04	4.58*E* + 04	5.58*E* + 04	4.08*E* + 04	
	Q75	4.58*E* + 04	6.17*E* + 04	6.83*E* + 04	5.92*E* + 04	
5	Median	5.42*E* + 04	5.58*E* + 04	7.17*E* + 04	5.92*E* + 04	0.34
	Q25	5.08*E* + 04	4.83*E* + 04	5.67*E* + 04	5.75*E* + 04	
	Q75	5.50*E* + 04	5.83*E* + 04	7.75*E* + 04	7.75*E* + 04	
6	Median	5.50*E* + 04	6.25*E* + 04	9.58*E* + 04	6.75*E* + 04	0.03
	Q25	4.33*E* + 04	5.67*E* + 04	8.25*E* + 04	4.92*E* + 04	
	Q75	5.58*E* + 04	7.33*E* + 04	1.03*E* + 05	6.75*E* + 04	
7	Median	6.42*E* + 04	5.67*E* + 04	9.50*E* + 04	6.00*E* + 04	0.04
	Q25	4.67*E* + 04	5.17*E* + 04	7.67*E* + 04	5.25*E* + 04	
	Q75	9.00*E* + 04	5.83*E* + 04	9.92*E* + 04	7.00*E* + 04	
8	Median	5.33*E* + 04	8.58*E* + 04	6.58*E* + 04	1.00*E* + 05	0.52
	Q25	3.83*E* + 04	7.75*E* + 04	5.75*E* + 04	6.25*E* + 04	
	Q75	7.17*E* + 04	8.58*E* + 04	1.13*E* + 05	1.00*E* + 05	
9	Median	7.33*E* + 04	6.58*E* + 04	8.92*E* + 04	6.08*E* + 04	0.72
	Q25	5.33*E* + 04	5.83*E* + 04	7.17*E* + 04	5.08*E* + 04	
	Q75	7.50*E* + 04	7.83*E* + 04	9.42*E* + 04	7.00*E* + 04	
10	Median	8.17*E* + 04	5.42*E* + 04	7.17*E* + 04	7.00*E* + 04	0.41
	Q25	6.25*E* + 04	4.83*E* + 04	7.08*E* + 04	5.83*E* + 04	
	Q75	8.25*E* + 04	9.92*E* + 04	8.83*E* + 04	8.83*E* + 04	
11	Median	7.17*E* + 04	5.75*E* + 04	8.25*E* + 04	7.83*E* + 04	0.73
	Q25	5.67*E* + 04	4.50*E* + 04	7.50*E* + 04	4.58*E* + 04	
	Q75	8.67*E* + 04	8.92*E* + 04	8.75*E* + 04	7.83*E* + 04	
12	Median	6.58*E* + 04	6.08*E* + 04	7.54*E* + 04	7.17*E* + 04	0.55
	Q25	5.50*E* + 04	3.92*E* + 04	4.67*E* + 04	4.00*E* + 04	
	Q75	7.50*E* + 04	8.08*E* + 04	1.07*E* + 05	7.42*E* + 04	
13	Median	5.58*E* + 04	6.67*E* + 04	1.13*E* + 05	6.75*E* + 04	0.06
	Q25	4.50*E* + 04	5.67*E* + 04	8.83*E* + 04	6.08*E* + 04	
	Q75	5.67*E* + 04	1.11*E* + 05	1.26*E* + 05	1.06*E* + 05	
14	Median	7.08*E* + 04	6.92*E* + 04	9.58*E* + 04	9.42*E* + 04	0.22
	Q25	5.17*E* + 04	5.83*E* + 04	7.83*E* + 04	6.42*E* + 04	
	Q75	7.58*E* + 04	8.50*E* + 04	1.00*E* + 05	1.00*E* + 05	

## Data Availability

All data used to support the findings of this study are included within the article.

## References

[1] Cekici A., Kantarci A., Hasturk H., Van Dyke T. E. (2014). Inflammatory and immune pathways in the pathogenesis of periodontal disease. *Periodontology 2000*.

[2] Nishida M., Grossi S. G., Dunford R. G., Ho A. W., Trevisan M., Genco R. J. (2000). Dietary vitamin C and the risk for periodontal disease. *Journal of periodontology*.

[3] Yu J., Tu Y. K., Tang Y. B., Cheng N. C. (2014). Stemness and transdifferentiation of adipose-derived stem cells using L-ascorbic acid 2-phosphate-induced cell sheet formation. *Biomaterials*.

[4] Li C. J., Sun L. Y., Pang C. Y. (2015). Synergistic protection of N-acetylcysteine and ascorbic acid 2-phosphate on human mesenchymal stem cells against mitoptosis, necroptosis and apoptosis. *Scientific Reports*.

[5] Gao Y., Han Z., Li Q. (2015). Vitamin C induces a pluripotent state in mouse embryonic stem cells by modulating microRNA expression. *The FEBS journal*.

[6] Esteban M. A., Pei D. (2012). Vitamin C improves the quality of somatic cell reprogramming. *Nature Genetics*.

[7] Chitsazi M., Faramarzie M., Sadighi M., Shirmohammadi A., Hashemzadeh A. (2017). Effects of adjective use of melatonin and vitamin C in the treatment of chronic periodontitis: a randomized clinical trial. *Journal of dental research, dental clinics, dental prospects*.

[8] Abou Sulaiman A. E., Shehadeh R. M. H. (2010). Assessment of total antioxidant capacity and the use of vitamin C in the treatment of non-smokers with chronic periodontitis. *Journal of periodontology*.

[9] Fawzy El-Sayed K. M., Paris S., Becker S. T. (2012). Periodontal regeneration employing gingival margin-derived stem/progenitor cells: an animal study. *Journal of clinical periodontology*.

[10] Fawzy El-Sayed K. M., Mekhemar M. K., Beck-Broichsitter B. E. (2015). Periodontal regeneration employing gingival margin-derived stem/progenitor cells in conjunction with IL-1ra-hydrogel synthetic extracellular matrix. *Journal of clinical periodontology*.

[11] Fawzy El-Sayed K. M., Dörfer C. E. (2016). Gingival mesenchymal stem/progenitor cells: a unique tissue engineering gem. *Stem Cells International*.

[12] Zhang Q., Shi S., Liu Y. (2009). Mesenchymal stem cells derived from human gingiva are capable of immunomodulatory functions and ameliorate inflammation-related tissue destruction in experimental colitis. *The Journal of Immunology*.

[13] Fawzy-El-Sayed K., Mekhemar M., Adam-Klages S., Kabelitz D., Dorfer C. (2016). TLR expression profile of human gingival margin-derived stem progenitor cells. *edicina oral, patologia oral y cirugia bucal*.

[14] Mekhemar M. K., Adam-Klages S., Kabelitz D., Dorfer C. E. (2018). Fawzy El-Sayed KM. TLR-induced immunomodulatory cytokine expression by human gingival stem/progenitor cells. *Cellular Immunology*.

[15] Ge S., Mrozik K. M., Menicanin D., Gronthos S., Bartold P. M. (2012). Isolation and characterization of mesenchymal stem cell-like cells from healthy and inflamed gingival tissue: potential use for clinical therapy. *Regenerative Medicine*.

[16] Zhang F., Si M., Wang H., Mekhemar M. K., Dorfer C. E., Fawzy El-Sayed K. M. (2017). IL-1/TNF-alpha inflammatory and anti-inflammatory synchronization affects gingival stem/progenitor cells’ regenerative attributes. *Stem Cells International*.

[17] Zhou L., Dorfer C. E., Chen L., Fawzy El-Sayed K. M. (2017). *Porphyromonas gingivalis* lipopolysaccharides affect gingival stem/progenitor cells attributes through NF-*κ*B, but not Wnt/*β*-catenin, pathway. *Journal of clinical periodontology*.

[18] Fawzy El‐Sayed K. M., Elahmady M., Adawi Z. (2019). The periodontal stem/progenitor cell inflammatory-regenerative cross talk: a new perspective. *Journal of Periodontal Research*.

[19] Fawzy El-Sayed K. M., Hein D., Dorfer C. E. (2019). Retinol/inflammation affect stemness and differentiation potential of gingival stem/progenitor cells via Wnt/*β*-catenin. *Journal of Periodontal Research*.

[20] Van Pham P., Tran N. Y., Phan N. L., Vu N. B., Phan N. K. (2016). Vitamin C stimulates human gingival stem cell proliferation and expression of pluripotent markers. *In vitro cellular & developmental biology Animal*.

[21] Fawzy El-Sayed K. M., Paris S., Graetz C. (2015). Isolation and characterisation of human gingival margin-derived STRO-1/MACS^+^ and MACS^−^ cell populations. *International journal of oral science*.

[22] Sidney L. E., Kirkham G. R., Buttery L. D. (2014). Comparison of osteogenic differentiation of embryonic stem cells and primary osteoblasts revealed by responses to IL-1*β*, TNF-*α*, and IFN-*γ*. *Stem cells and development*.

[23] Fawzy El-Sayed K. M., Klingebiel P., Dorfer C. E. (2016). Toll-like receptor expression profile of human dental pulp stem/progenitor cells. *Journal of Endodontics*.

[24] Ng T. K., Huang L., Di Cao Y. W.-Y. Y. (2015). Cigarette smoking hinders human periodontal ligament-derived stem cell proliferation, migration and differentiation potentials. *Scientific Reports*.

[25] Mahmood M., Li Z. G., Casciano D. (2011). Nanostructural materials increase mineralization in bone cells and affect gene expression through miRNA regulation. *Journal of Cellular and Molecular Medicine*.

[26] Sun J. Q., Ye X., Xie M. H., Ye J. P. (2016). Induction of triglyceride accumulation and mitochondrial maintenance in muscle cells by lactate. *Scientific Reports*.

[27] Mekhemar M., Tolle J., Dorfer C., Fawzy E.-S. K. (2020). TLR3 ligation affects differentiation and stemness properties of gingival mesenchymal stem/progenitor cells. *Journal of Clinical Periodontology*.

[28] Fawzy El-Sayed K. M., Dorfer C. E. (2017). Animal models for periodontal tissue engineering: a knowledge-generating process. *Tissue engineering Part C, Methods*.

[29] Silva T. A., Garlet G. P., Fukada S. Y., Silva J. S., Cunha F. Q. (2007). Chemokines in oral inflammatory diseases: apical periodontitis and periodontal disease. *Journal of Dental Research*.

[30] Lee J. S., Lee J. B., Cha J. K. (2017). Chemokine in inflamed periodontal tissues activates healthy periodontal-ligament stem cell migration. *Journal of clinical periodontology*.

[31] Rubin M. B. (1984). Vitamins and wound healing. *Plastic Surgical Nursing*.

[32] Chapple I. L. C. (1997). Reactive oxygen species and antioxidants in inflammatory diseases. *Journal of clinical periodontolog.*.

[33] Alagl A. S., Bhat S. G. (2015). Ascorbic acid: new role of an age-old micronutrient in the management of periodontal disease in older adults. *Geriatrics & gerontology international*.

[34] Majewicz J., Rimbach G., Proteggente A. R., Lodge J. K., Kraemer K., Minihane A. M. (2005). Dietary vitamin C down-regulates inflammatory gene expression in apoE4 smokers. *Biochemical and biophysical research communications*.

[35] Tomofuji T., Ekuni D., Sanbe T. (2009). Effects of vitamin C intake on gingival oxidative stress in rat periodontitis. *Free Radical Biology and Medicine*.

[36] Gao Y., Yang L., Chen L. (2013). Vitamin C facilitates pluripotent stem cell maintenance by promoting pluripotency gene transcription. *Biochimie*.

[37] Tomar G. B., Srivastava R. K., Gupta N. (2010). Human gingiva-derived mesenchymal stem cells are superior to bone marrow-derived mesenchymal stem cells for cell therapy in regenerative medicine. *Biochemical and biophysical research communication.*.

[38] Fournier B. P. J., Ferre F. C., Couty L. (2010). Multipotent progenitor cells in gingival connective tissue. *Tissue engineering Part A*.

[39] Jin S. H., Lee J. E., Yun J. H., Kim I., Ko Y., Park J. B. (2015). Isolation and characterization of human mesenchymal stem cells from gingival connective tissue. *Journal of Periodontal Research*.

[40] Gao Y., Zhao G., Li D., Chen X., Pang J., Ke J. (2014). Isolation and multiple differentiation potential assessment of human gingival mesenchymal stem cells. *International journal of molecular sciences*.

[41] Dominici K. L. B., Mueller I., Slaper-Cortenbach I. (2006). Minimal criteria for defining multipotent mesenchymal stromal cells. The International Society for Cellular Therapy position statement. *Cytotherapy*.

[42] Al Bahrawy M., Ghaffar K., Gamal A., El-Sayed K., Iacono V. (2020). Effect of inflammation on gingival mesenchymal stem/progenitor cells’ proliferation and migration through microperforated membranes: an in vitro study. *Stem Cells International*.

[43] Zhou L. L., Liu W., Wu Y. M., Sun W. L., Dorfer C. E., Fawzy El-Sayed K. M. (2020). Oral mesenchymal stem/progenitor cells: the immunomodulatory masters. *Stem Cells International*.

[44] Rasmussen M. L., Ortolano N. A., Romero-Morales A. I., Gama V. (2018). Wnt Signaling and Its Impact on Mitochondrial and Cell Cycle Dynamics in Pluripotent Stem Cells. *Genes*.

[45] Liu N., Shi S., Deng M. (2011). High levels of *β*-catenin signaling reduce osteogenic differentiation of stem cells in inflammatory microenvironments through inhibition of the noncanonical Wnt pathway. *Journal of Bone and Mineral Research*.

[46] Kong X., Liu Y., Ye R. (2013). GSK3*β* is a checkpoint for TNF-*α*-mediated impaired osteogenic differentiation of mesenchymal stem cells in inflammatory microenvironments. *Biochimica et Biophysica Acta*.

[47] Chiquet M., Katsaros C., Kletsas D. (2015). Multiple functions of gingival and mucoperiosteal fibroblasts in oral wound healing and repair. *Periodontology 2000*.

[48] Potdar P. D., D'Souza S. B. (2010). Ascorbic acid induces *in vitro* proliferation of human subcutaneous adipose tissue derived mesenchymal stem cells with upregulation of embryonic stem cell pluripotency markers Oct 4 and SOX 2. *Human cell*.

[49] Tomasello L., Mauceri R., Coppola A. (2017). Mesenchymal stem cells derived from inflamed dental pulpal and gingival tissue: a potential application for bone formation. *Stem cell research & therapy*.

[50] Hore T. A., von Meyenn F., Ravichandran M. (2016). Retinol and ascorbate drive erasure of epigenetic memory and enhance reprogramming to naive pluripotency by complementary mechanisms. *Proceedings of the National Academy of Sciences of the United States of America*.

[51] Hore T. A. (2017). Modulating epigenetic memory through vitamins and TET: implications for regenerative medicine and cancer treatment. *Epigenomics*.

[52] Kim J. H., Kim W. K., Sung Y. K. (2014). The molecular mechanism underlying the proliferating and preconditioning effect of vitamin C on adipose-derived stem cells. *Stem Cells and Development*.

[53] Choi K. M., Seo Y. K., Yoon H. H. (2008). Effect of ascorbic acid on bone marrow-derived mesenchymal stem cell proliferation and differentiation. *Journal of Bioscience and Bioengineering*.

[54] Liu Y., Wang L., Kikuiri T. (2011). Mesenchymal stem cell-based tissue regeneration is governed by recipient T lymphocytes via IFN-*γ* and TNF-*α*. *Nature medicine*.

[55] Carinci F., Pezzetti F., Spina A. M. (2005). Effect of Vitamin C on pre-osteoblast gene expression. *Archives of oral biology*.

[56] Ishikawa S., Iwasaki K., Komaki M., Ishikawa I. (2004). Role of ascorbic acid in periodontal ligament cell differentiation. *Journal of periodontology*.

